# A RE-AIM Framework Analysis of DNA-Based Population Screening: Using Implementation Science to Translate Research Into Practice in a Healthcare System

**DOI:** 10.3389/fgene.2022.883073

**Published:** 2022-05-25

**Authors:** Laney K. Jones, Natasha T. Strande, Evan M. Calvo, Jingheng Chen, Gabriela Rodriguez, Cara Z. McCormick, Miranda L. G. Hallquist, Juliann M. Savatt, Heather Rocha, Marc S. Williams, Amy C. Sturm, Adam H. Buchanan, Russell E. Glasgow, Christa L. Martin, Alanna Kulchak Rahm

**Affiliations:** ^1^ Genomic Medicine Institute, Geisinger, Danville, PA, United States; ^2^ Heart and Vascular Institute, Geisinger, Danville, PA, United States; ^3^ Autism & Developmental Medicine Institute, Geisinger, Danville, PA, United States; ^4^ Department of Human Genetics, Graduate School of Public Health, University of Pittsburgh, Pittsburgh, PA, United States; ^5^ University of Colorado Anschutz Medical Campus, Aurora, CO, United States

**Keywords:** DNA-based population screening, implementation science, healthcare system, RE-AIM, genetics, MyCode

## Abstract

**Introduction:** DNA-based population screening has been proposed as a public health solution to identify individuals at risk for serious health conditions who otherwise may not present for medical care. The clinical utility and public health impact of DNA-based population screening is a subject of active investigation. Geisinger, an integrated healthcare delivery system, was one of the first healthcare systems to implement DNA screening programs (MyCode Community Health Initiative (MyCode) and clinical DNA screening pilot) that leverage exome data to identify individuals at risk for developing conditions with potential clinical actionability. Here, we demonstrate the use of an implementation science framework, RE-AIM (Reach, Effectiveness, Adoption, Implementation and Maintenance), to conduct a post-hoc evaluation and report outcomes from these two programs to inform the potential impact of DNA-based population screening.

**Methods:** Reach and Effectiveness outcomes were determined from the MyCode research program, while Adoption and Implementation outcomes were measured using the clinical DNA screening pilot. Reach was defined as the number of patients who were offered and consented to participate in MyCode. Effectiveness of DNA screening was measured by reviewing MyCode program publications and synthesizing findings from themes. Adoption was measured by the total number of DNA screening tests ordered by clinicians at the clinical pilot sites. Implementation was assessed by interviewing a subset of clinical pilot clinicians about the deployment of and recommended adaptations to the pilot that could inform future program dissemination.

**Results:** *Reach*: As of August 2020, 68% (215,078/316,612) of individuals approached to participate in the MyCode program consented. Effectiveness: Published evidence reported from MyCode demonstrates that DNA screening identifies at-risk individuals more comprehensively than clinical ascertainment based on phenotypes or personal/family history. *Adoption*: From July 2018 to June 2021, a total of 1,026 clinical DNA screening tests were ordered by 60 clinicians across the three pilot clinic sites. *Implementation*: Interviews with 14 clinicians practicing at the pilot clinic sites revealed motivation to provide patients with DNA screening results and yielded future implementation strategies.

**Conclusion:** The RE-AIM framework offers a pragmatic solution to organize, analyze, and report outcomes across differently resourced and designed precision health programs that include genomic sequencing and return of clinically actionable genomic information.

## Introduction

DNA-based population screening of unselected individuals for disease-causing genomic variants has been proposed as a method for ascertaining those at risk for serious health conditions who may not otherwise be identified. This distinction of unselected individuals is critical to exploring DNA-based population screening as it refers to the system-wide selection or screening of individuals without regard to underlying risk, clinical features, or family history that may indicate hereditary risk or disease (indication-based identification) (Carey et al., 2016; Abul-Husn et al., 2019; Abul-Husn et al., 2021). Such screening has the benefit of identifying individuals with actionable genetic changes (Kalia et al., 2017) prior to diagnosis based on symptoms; symptoms which are typically the impetus for indication-based genetic testing. By identifying individuals earlier, appropriate medical action for treatment and prevention can be taken. Another benefit is the potential to overcome inequities and health disparities currently seen in indication-based identification and testing (Jakuboski et al., 2022). Several healthcare systems have initiated DNA-based population screening programs that may consist of research biobanks and/or DNA screening pilot programs (Williams, 2022). Early results from these programs demonstrate effectiveness in ascertaining individuals carrying genomic risk variants by improving risk management and facilitating early diagnoses of severe diseases (Grzymski et al., 2020; Williams, 2022). However, a key issue limiting the implementation of these DNA-based population screening programs into routine clinical care is the critical need for additional evidence demonstrating clinical utility (Murray et al., 2019).

Ongoing evidence gaps recognized in DNA-based population screening include questions about which genes—and variants within those genes—to screen, best practices for disclosing results to individuals and clinicians, short- and long-term outcomes of returning genomic information, when to perform screening, settings and care models for screening, costs and cost-effectiveness of screening, and whether screening mitigates or exacerbates health disparities. Many of these gaps relate to clinical utility, defined by the Centers for Disease Control and Prevention (CDC) as “whether genetic testing results in measurable improvement in health or improves management of patients” (Haddow et al., 2004; Murray et al., 2019; Office of Science, 2021).

In the traditional research model, evidence gaps are addressed through studies of efficacy and effectiveness conducted in narrowly defined populations or organizational contexts (Chambers et al., 2016). This has led to the general observation that it takes an average of 17 years to translate a fraction of such research into clinical care (Balas and Boren, 2000). The field of implementation science has evolved to help shorten the time to implementation of effective interventions by understanding the multi-level, complex issues inherent in the implementation, adoption, and maintenance of research evidence in health care policy and practice (Holtrop et al., 2018a; Chambers, 2018). Implementation science focuses on evaluating use and effectiveness under typical (real-world, non-controlled) conditions (Holtrop et al., 2018a) by leveraging theories, models, and frameworks for program planning, implementation, evaluation, and maintenance (Nilsen, 2015; Brownson, 2017). Due to the rapid generation of data and the need to expediate the translation of learnings from multiple contexts such as observational studies, clinical trials, and pilot programs, calls for incorporation of implementation science methodologies into the fields of genomics and precision health have been made (Chambers, 2018; Ginsburg et al., 2021; Sperber et al., 2021).

The RE-AIM framework (Reach, Effectiveness, Adoption, Implementation, and Maintenance) is an implementation science framework that addresses the research-practice gap by including evaluation of outcomes beyond efficacy and effectiveness to better identify translation potential and public health impact (Glasgow et al., 1999; Glasgow et al., 2019). RE-AIM emphasizes both internal and external validity by evaluating outcomes associated with the five dimensions in the framework acronym. RE-AIM is ideal for pragmatic contexts and facilitates evaluation of impact at the individual (reach/effectiveness) and institutional (adoption/implementation) levels simultaneously, since multi-level impact is critical for both translation and broader public health benefit (Glasgow et al., 1999; Nilsen, 2015; Brownson, 2017; Glasgow and Estabrooks, 2018; Glasgow et al., 2019). Over the last two decades, RE-AIM has been used extensively in other contexts, yet it is only beginning to be applied to precision health (Glasgow et al., 2019; Jones et al., 2021b; Blazer et al., 2021; Kim et al., 2021; Miller et al., 2021; Sperber et al., 2021). In this study, we demonstrate the use of RE-AIM to conduct a post-hoc evaluation and report outcomes from two DNA screening programs at Geisinger with the goal of generating evidence needed for systematic implementation of DNA-based population screening.

## Materials and Methods

### Study Setting

Geisinger, an integrated health system serving over two million individuals in rural Pennsylvania, is an innovator in exploring DNA-based population screening approaches (Carey et al., 2016; Schwartz et al., 2018; Schmidlen et al., 2019; Savatt et al., 2020). Approximately one-third of individuals receiving care in the system are also insured by the Geisinger Health Plan, creating the ideal environment for piloting innovations in care delivery to improve health outcomes (Steele and Feinberg, 2017). Two of Geisinger’s precision health programs are described above with key aspects of each program highlighted in Table 1.

**TABLE 1:** Key characteristics of two Geisinger DNA screening programs.

Characteristics	MyCode^®^ community health initiative (research)	Clinical DNA screening pilot
Purpose	Return clinically actionable confirmed findings from research exome sequences to MyCode participants	Return subset of clinically actionable findings from clinically generated exome sequences to unselected patients at participating clinics
Implementation context	Geisinger population and MyCode participants	Patients receiving care at specific ambulatory clinics (primary and specialty care)
Who offers/delivers the program	Precision health associates (consenters), GCs, genetic counseling assistants funded through the GSC program	Clinicians at select sites as part of clinical practice
Screening model	Opportunistic	Proactive
Genes screened	ACMG SF v2.0 + *HFE* (c.845G > A, p.C282Y homozygotes)	ACMG SF v2.0
Who discloses results	GSC GCs	• GCs modeling the GSC disclosure process (positive results)
		• Ordering clinician *via* letter (negative results)
Timeline to result return	6 months–2 years based on sample batch size	6–8 weeks

ACMG, American College of Medical Genetics and Genomics; GC, genetic counselor; GSC, genomic screening and counseling.

#### MyCode Community Health Initiative (MyCode)

In-depth descriptions of Geisinger’s MyCode program have been published elsewhere (Carey et al., 2016; Schwartz et al., 2018; Williams, 2019; Williams et al., 2018b; Kelly et al., 2021; Dewey et al., 2016; MyCode scorecard [Online], n.a). Relevant to the analyses presented here, Geisinger launched MyCode in 2007 to create a biobank of serum, blood, and DNA samples for health discovery research (Carey et al., 2016). The overall aim is to develop methods that will enable identification of individuals’ unique biological, environmental, and social influences on health and promote care tailored to individual health risks (Williams et al., 2018b; Williams, 2019). In 2014, MyCode initiated exome sequencing and SNP genotyping using DNA samples through the DiscovEHR collaboration with the Regeneron Genetics Center to uncover novel genetic associations with disease and therapeutic targets (Dewey et al., 2016; Kelly et al., 2021). In anticipation of exome sequencing, MyCode amended its consent in 2013 to allow disclosure of clinically actionable findings to participants (Carey et al., 2016; Kelly et al., 2021). Any Geisinger patient is eligible to participate in MyCode and can consent in-person when they present for care or *via* the patient portal in the electronic health record (EHR). Consent documents are currently available in English and Spanish. MyCode participants who consented prior to 2013 are contacted by study staff to re-consent for DNA screening and potential return of information. As of February 2022, >300,000 Geisinger patients have consented to MyCode, >207,000 have provided samples, >184,000 have had exome sequencing and genotyping completed, and >3,100 have received a clinically actionable genetic result (MyCode scorecard [Online], n.a).

The MyCode Genomic Screening and Counseling (GSC) program was added in 2015 to identify and clinically confirm actionable genomic risk results for disclosure to patient-participants and their clinicians (Williams et al., 2018b; Schwartz et al., 2018). When MyCode exome sequence data reveals a pathogenic or likely pathogenic (P/LP) variant in a gene returned through MyCode, a clinically collected sample retained in the MyCode CLIA-certified repository is sent for clinical confirmation and reporting of the variant in a CLIA-certified genetics laboratory. The list of genes included for DNA screening through MyCode was developed based on several resources including to the American College of Medical Genetics and Genomics (ACMG) secondary findings list, as previously described, and is regularly reviewed and updated by research and clinical stakeholders based on current evidence (Schwartz et al., 2018; Kelly et al., 2021). The current list of genes includes those on the ACMG secondary findings v2.0 list (Kalia et al., 2017) in addition to biallelic variants in the *HFE* gene leading to the C282Y amino acid substitution (Kelly et al., 2021). Benign/likely benign variants and variants of uncertain significance (VUS) are not reported to MyCode participants. After CLIA confirmation of the result, the GSC program process includes 1) depositing the laboratory report with genetic test results into the patient-participant’s EHR, 2) notifying the patient-participant’s primary care clinician through the EHR (for Geisinger clinicians) or *via* alternative methods for external clinicians, 3) three phone call/patient portal attempts to disclose the result and recommend a complimentary genetic counseling visit, and 4) mailing of a packet with the result to the patient-participant (Schwartz et al., 2018).

#### Clinical DNA Screening Pilot Program

In 2018, Geisinger launched a clinical DNA screening pilot program in select ambulatory care settings to evaluate the integration of DNA screening into routine healthcare (Geisinger, 2018). The clinical DNA screening test uses an exome sequencing backbone to screen for P/LP variants in the genes on the ACMG secondary findings version 2.0 list (Kalia et al., 2017). Positive screen results (P/LP variants) are disclosed to patients by a genetic counselor utilizing a modified version of the MyCode GSC program disclosure protocols; negative results are disclosed by a letter to the patient. Given the pivotal role that primary care providers play in preventive care, the clinical DNA screening pilot program was initiated to engage primary care in delivering genomic screening as a part of routine primary care practice.

The program is a system initiative that was initially implemented as a pragmatic proof-of-concept clinical pilot at 3 clinics selected based on location (one clinic per service region: central, northeast, west) and clinical interest. Clinical tools (EHR-based ordering and documenting templates) and educational information developed in collaboration with clinical partners were provided to each pilot clinic site upon program implementation at the site. All clinicians at the three clinical pilot sites can order the clinical DNA screening test for any adult individual seen irrespective of disease indications.

### Study Design

We conducted a post-hoc program evaluation of data generated from the MyCode research program and the clinical DNA-screening pilot program using mixed-methods and the RE-AIM framework as adapted based on guidance in Glasgow & Estabrooks for the pragmatic gathering of data and evaluation of relevant outcomes (Glasgow and Estabrooks, 2018). This evaluation was deemed not research by the Geisinger Institutional Review Board.

Table 2 describes how the two programs inform the potential for implementation of population-based DNA screening by RE-AIM dimension and the data/method utilized to inform results. The MyCode research program provides the context most similar to real-world conditions for patient interest in and effectiveness of broad-scale population screening were it to be made available to all individuals in a health system. Therefore, Reach and Effectiveness were evaluated through the MyCode research program using MyCode consent database (Reach) and publication review (Effectiveness) to understand the potential willingness of individuals to participate in a research program that discloses health-related genomic results to participants and the impact of returning genomic information to individuals on health outcomes. As a system initiative based in primary care, the clinical DNA screening pilot program demonstrates how such a program might look in clinical practice and created an ideal natural experiment to measure Adoption and Implementation of DNA-based population screening by eligible clinicians under real-world conditions. EHR data (Adoption) and qualitative interviews (Implementation) were conducted to understand adoption variability and clinician views of offering and ordering a DNA screening test as part of routine healthcare. Maintenance was not assessed in this evaluation but guidance for how this dimension may be measured is included in Table 2.

**TABLE 2:** RE-AIM dimensions with standard definitions, adapted definitions, associated Geisinger DNA screening programs, and data sources.

Dimensions	Definition	DNA-based population screening definition	Program	Data sources
Reach	The absolute number, proportion, and representativeness of individuals willing to participate in a program	The number, proportion, and representativeness of individuals willing to participate in a DNA-based population program that returns genomic information	MyCode (research)	MyCode consent database
Effectiveness	The impact of an intervention on important individual outcomes, including potential negative effects, and broader impact including quality of life and economic outcomes; and variability across subgroups (generalizability or heterogeneity of effects)	The impact of returning clinically relevant genetic results to individuals on medical outcomes, psychological and quality of life outcomes, and economic outcomes, including negative effects. Variability across subgroups and including health disparities	MyCode (research)	Review of published MyCode literature
Adoption	The absolute number, proportion, and representativeness of people who deliver the program and who are willing to initiate a program	The number of clinical genomic screening tests ordered at pilot sites	Clinical DNA screening pilot	EHR
Implementation	Any adaptations made to interventions and implementation strategies	Suggested adaptations to the current clinical pilot to inform future program dissemination	Clinical DNA screening pilot	Semi-structured interviews with clinicians
Maintenance	(setting level) the extent to which a program or policy becomes institutionalized or part of the routine organizational practices and policies, and adaptations made to achieve maintenance	(setting level) extent to which MyCode and clinical pilot programs become routine/institutionalized	Not yet assessed	Not applicable
		(individual level) long term impact (e.g., longitudinal effectiveness, adherence to guidelines) of returning clinically relevant genetic information on individual health outcomes		
	(individual level) the long-term effects of a program on outcomes after a program is completed			

### Definition, Data Collection, and Outcomes Analysis Methods Per RE-AIM Dimension

#### Reach

Reach was defined as the number of individuals who consented or re-consented to MyCode after 2013 (when updates to consenting allowed for disclosure of results) over the number of individuals approached to participate in the program. Representativeness (a critical component of Reach) of the population reached by MyCode was also explored. Representativeness of MyCode patient-participants was compared to non-participants (individuals who have declined or withdrawn participation or have not yet re-consented) and compared to the system’s general patient population (inclusive of all individuals who have received care at Geisinger regardless of whether they are insured by Geisinger or have a Geisinger primary care clinician).

MyCode consent data is stored in a MyCode consent database. Information from this database from February 2007 to August 2020 were reviewed for individuals approached to participate in MyCode. Demographic data available from the EHR included current age, sex, race, ethnicity, 3-digit level zip code, primary care clinician (Geisinger or non-Geisinger), comorbidity index, and health insurance type. Descriptive characteristics were reported using means and medians and comparisons of categorical variables between groups were performed by Chi-squared test or Z-test for proportions. Non-normal continuous variables were compared using Wilcoxon rank sum test. All statistical analysis was performed in R (Vienna, Version 4.0.2).

#### Effectiveness

Effectiveness was defined as the clinical impact of returning clinically relevant genetic results to individuals. Since multiple analyses have already been conducted and published, effectiveness was evaluated by conducting a review of this published MyCode literature. Thirteen peer-reviewed publications have reported MyCode outcomes related to Effectiveness of DNA-based population screening from program initiation (2007) to 2021. Data extracted from these studies included genetic condition, study sample size, and key findings. Studies were organized and coded for the following thematic outcomes determined by the study team to represent Effectiveness: screen positive detection rate (proportion of eligible participants with a clinically confirmed P/LP variant in a gene of interest), ascertainment of at-risk individuals *via* DNA screening compared to clinical ascertainment, rate of relevant genetic disease, impact of genetic results disclosure on medical management, post-disclosure disease diagnoses attributed to DNA screening, and costs and cost-effectiveness. Coding was conducted by two raters, with discrepancies reviewed and resolved by the senior author. A brief description of each thematic area and relevance to population-based DNA screening is described below:

• Screen positive detection rate of actionable genomic variants in unselected populations: Demonstrating the P/LP variant rate related to a condition in an unselected population is an important indicator of how many at-risk individuals in a population remain unidentified or undiagnosed without DNA-based population screening.
• Ascertainment of at-risk individuals *via* DNA screening compared to clinical ascertainment: Comparing the number of individuals with P/LP variants, but unrecognized prior to DNA screening, to clinical ascertainment as a key indicator of programmatic effectiveness.
• Rate of relevant genetic disease: Understanding the rate of relevant disease among unselected individuals found to have an actionable variant can inform recommendations for managing their disease risks.
• Impact of disclosure on medical management: For population DNA screening to have the intended public health benefit, clinicians and patients must adhere to recommended medical management intended to reduce condition-specific morbidity and mortality. Identification of multilevel barriers to and facilitators of recommended management can inform interventions to improve management.
• New clinical diagnoses post-disclosure: A goal of population-based DNA screening is to impact the condition-related health outcomes of the individual identified with a P/LP variant for the condition.
• Cost and cost-effectiveness: Cost-burden on a healthcare system or patients and cost-effectiveness of population-based DNA screening is a reported barrier to implementation and an important factor for sustainability.

#### Adoption

For this post hoc evaluation of DNA-based population screening, Adoption was defined as the number of the clinical DNA screening tests ordered by clinicians at the clinical pilot sites, as determined by review of programmatic data. Provider type (attending, resident, Fellow, etc.) was collected to describe representativeness. Due to the clinical pilot program implementation that made the test available to all clinicians in the pilot clinic and because of the fluctuation in attending clinicians and trainees in pilot sites over time, the percentage of clinicians ordering the clinical DNA screening test (proportion/percent adopted) could not be accurately determined.

#### Implementation

Implementation was assessed by conducting semi-structured interviews among a subset of clinical pilot clinicians about the deployment of and recommended adaptations to the pilot that could inform future program improvement and dissemination.

Clinicians, including physicians (attendings, residents, and fellows), nurse practitioners, and physician assistants were invited to participate in the interviews. Clinicians were recruited through email using a purposive sampling strategy based on clinic and number of clinical DNA screening tests ordered during the pilot implementation (including clinicians that did not order the test) to ensure representation across pilot clinics (location-central, northeast, or west) and adoption (no tests ordered, 1–10 tests ordered, 11–20 tests ordered, 21–30 tests ordered, over 100 tests ordered). All interviews were conducted using a semi-structured interview guide to explore implementation aspects of the pilot clinical DNA screening program and inform future program dissemination. Questions were designed to explore attitudes towards clinical DNA screening in primary care, why or for whom they ordered testing for and experience with testing and results (if ordered), fit with clinical workflow, confidence in understanding and using the test information, experience with and opinion of EHR tools provided, and recommendations to improve the program and processes (See Supplementary Material S1 for interview guide).

A rapid qualitative analysis using a framework method was employed (Bryman and Burgess, 1994; Gale et al., 2013). Two research staff members reviewed interview summaries and full transcripts under the guidance of the first author to define emergent themes and identify supportive quotations. Emergent themes were finalized through discussion with the first author and coding accuracy was achieved through constant comparison with the first author (Beebe, 2001). Discrepancies and uncertainties with themes identified and coded quotations were resolved by additional expert consultation with the senior author. Prior to finalizing, all results were reviewed with clinical screening pilot program staff and other study team members.

## Results

### Reach

Approximately two million individuals receive care within the Geisinger system. All have the potential to participate in MyCode by enrolling through the patient portal or when receiving care in a Geisinger facility. Of the 316,612 individuals approached to participate in the MyCode research program, 215,078 individuals had consented or re-consented after 2013 (when updates to consenting allowed for disclosure of results) as of August 2020 (68% participation rate). To evaluate the representativeness of MyCode participants, we compared those consented to receive results to individuals who actively declined to participate (78,372), withdrew consent (3,577) or have not yet re-consented to receive results (18,355) and to the general Geisinger population (2,072,639) (Table 3). There were statistically significant differences in demographic characteristics between individuals on a return-eligible consent (willing to participate) compared to those who were not eligible to receive results (declined, withdrew, or have not yet updated their consent) and to the general Geisinger population. Individuals who consented to receive results were younger than those not eligible to receive results, but were older than the overall Geisinger population (*p* < 2.2 × 10^−16^). They were also more likely to be female (*p* < 0.0001), White (*p* < 2.2 × 10^−16^), non-Hispanic (*p* < 2.2 × 10^−16^), have a Geisinger primary care physician (PCP) (*p* < 0.0001), have Geisinger Health Plan insurance (*p* < 2.2 × 10^−16^), and have a higher Charlson Comorbidity Index (*p* < 2.2 × 10^−16^) than both comparator populations.

**TABLE 3:** Characteristics of MyCode participants and general Geisinger population.

	MyCode participants who consented or re-consented after 2013 (*N* = 2,15,078)	MyCode participants who declined or withdrew or have not reconsented after 2013 (*N* = 1,00,314)	*p*-value^a^	General Geisinger population (*N* = 20,72,639)	*p*-value^b^
Age, median [IQR]	55 [38, 68]	57 [39, 71]	*p* < 2.2 × 10^−16^	40 [20, 62]	*p* < 2.2 × 10^−16^
Sex, n (%)					
Female	1,28,149 (59.6)	60,456 (60.3)	*p* < 0.0001	10,79,082 (52.1)	*p* < 2.2 × 10^−16^
Male	86,928 (40.4)	39,850 (39.7)		9,93,557 (47.9)	
Unknown	1 (0.0)	8 (0.0)			
Race, n (%)					
White/European ancestry	2,06,102 (95.8)	94,487 (94.2)	*p* < 2.2 × 10^−16^	18,76,010 (90.5)	*p* < 2.2 × 10^−16^
Black/African ancestry	5,771 (2.7)	3,795 (3.8)		1,09,164 (5.3)	
Native American	278 (0.1)	132 (0.1)		2,995 (0.1)	
Asian or Pacific Islander	1,516 (0.7)	1,515 (1.5)		36,894 (1.8)	
Unknown/other	1,411 (0.7)	385 (0.4)		47,576 (2.3)	
Ethnicity, n (%)					
Hispanic/Latinx	6,284 (2.9)	3,572 (3.6)	*p* < 2.2 × 10^−16^	1,07,788 (5.2)	*p* < 2.2 × 10^−16^
Not Hispanic/Latinx	2,06,776 (96.1)	94,725 (94.4)		—	
Unknown	2018 (0.9)	2017 (2.0)		—	
Have a Geisinger PCP, n (%)	1,32,652 (61.7)	60,428 (60.2)	*p* < 0.0001	5,94,847 (28.7)	*p* < 2.2 × 10^−16^
Insured with GHP, n (%)	82,926 (38.6)	34,240 (34.1)	*p* < 2.2 × 10^−16^	4,51,835 (21.8)	*p* < 2.2 × 10^−16^
CCI, median [IQR]	2 [0, 4]	2 [0, 4]	*p* < 2.2 × 10^−16^	0	*p* < 2.2 × 10^−16^

PCP, primary care provider; GHP, Geisinger health plan; CCI, Charlson comorbidity index; IQR, interquartile range.

a Comparison between MyCode screening population and control population. Chi-squared test was performed for categorical variables with multiple levels (Sex, Race, and Ethnicity). Z-test for two proportions was used for categorical variables with two levels (%Geisinger PCP, %GHP). Two-sample Wilcoxon test was used for comparing the medians for continuous variables (Age and CCI).

b Comparison between MyCode screening population and Geisinger population. Chi-squared test was performed for categorical variables with multiple levels (Sex and Race). Z-test for one proportion was used for logistical variables or categorical variables with two levels (Sex, % Hispanic/Latinx, %Geisinger PCP, %GHP). One-sample Wilcoxon test was used for non-normal continuous variables (Age and CCI), treating the medians of the general Geisinger population as the population median.

### Effectiveness

Table 4 provides detail on the multiple levels (population, individual, system) addressed by each identified thematic area relevant population-based DNA screening and the number of publications relevant to each theme at the time of this analysis. Outcomes related to themes of interest were extracted and summarized (Supplementary Material S2). Overall, our published results thus far indicate that DNA screening identifies at-risk individuals more comprehensively than clinical ascertainment based on phenotypes or personal/family history, that disclosing this information can have positive impact on individual medical management and diagnostic outcomes, and that costs and cost-effectiveness in different contexts are important to assess.

**TABLE 4:** Program review effectiveness construct results reported by clinical utility-associated thematic purpose.

Effectiveness-related themes	Level	Definition	Example	Number of publications to date	References
Screen positive detection rate of actionable genetic variants in unselected populations	Population	Defining the number with P/LP genetic variants	Reporting within the population on the number of individuals with P/LP genetic variants	5	(Kelly et al., 2021; Abul-Husn et al., 2016; Manickam et al., 2018; Carruth et al., 2021; Carruth et al., 2019)
Ascertainment of at-risk individuals *via* DNA screening compared to clinical ascertainment	Individual patient	Defining the number of individuals with P/LP variants and clinical phenotype that has not been previously identified	Have phenotype but were unrecognized to have the condition until receipt of the genetic information	5	(Buchanan et al., 2020; Buchanan et al., 2018; Manickam et al., 2018; Jones et al., 2021a)
Rate of relevant genetic disease	Individual patient	Comparing phenotypes of individuals with P/LP for the condition with individuals with only a clinical diagnosis	Clinical vs. genetic diagnosis of a condition	5	(Buchanan et al., 2020; Abul-Husn et al., 2016; Manickam et al., 2018; Carruth et al., 2019; Carruth et al., 2021)
Impact of disclosure on medical management	Individual patient	Reported on data congruency with desired outcome or guideline-based recommendation	Reporting on number of participants who would have been picked up on family history screening Number of participant adherent to guideline-based recommendations after receiving results	5	(Buchanan et al., 2018; Buchanan et al., 2020; Hao et al., 2020; Jones LK et al., 2018; Jones et al., 2020)
New clinical diagnoses post-disclosure	Individual patient	Medical follow-up prompted by the knowledge/return of the genomic information led directly to a diagnosis related to the variant (e.g., an ovarian cancer diagnosed) or a clinical manifestation of the diseases (e.g., aortic dilation identified after a Marfan variant returned)	Case reports or counts of new diagnoses reported post return of genetic result that can be linked to the return of the genomic information to the individual (e.g., are a direct result of medical follow-up specifically attributed to the result returned)	4	(Buchanan et al., 2020; Buchanan et al., 2018; Jones LK et al., 2018; Carruth et al., 2021)
Cost and cost effectiveness	Population or system	Reporting on costs per patients of genetic sequencing in a population	Quality adjusted life years of a genetic sequencing program (usually modeling papers)	3	(Hao et al., 2020; Guzauskas et al., 2020; Guzauskas GF et al., 2022)

P/LP, pathogenic or likely pathogenic.

#### Screen Positive Detection Rate of Actionable Genomic Variants in Unselected Populations

We found an overall detection rate of 2.6% for P/LP variants in the 60 genes screened by MyCode from 130,048 exomes screened at that time (Kelly et al., 2021). Thus far, MyCode data have reported P/LP variant detection rates in unselected individuals for familial hypercholesterolemia (FH)-related variants (1 in 222) (Abul-Husn et al., 2016) and hereditary breast and ovarian cancer (HBOC)-related variants (1 in 180) (Manickam et al., 2018). For arrhythmogenic cardiomyopathy (ACM), an inherited heart condition associated with sudden cardiac death, particularly in the young, MyCode data indicate a P/LP variant rate between 1 in 435 (Carruth et al., 2021) and 1 in 714 (Carruth et al., 2019), depending on the review criteria applied.

#### Ascertainment Of At-Risk Individuals *Via* DNA Screening Compared To Clinical Ascertainment

Based on EHR review, only 14%–20% of MyCode patient-participants had a clinical laboratory report documenting their genomic variant prior to identification through MyCode (Kelly et al., 2021). During the period under study, three genetic conditions were recognized by the CDC as having evidence for potential reduction in morbidity and mortality when identified through population DNA screening (Centers for Disease Control and Prevention, 2014). These conditions—HBOC syndrome (associated with *BRCA1* and *BRCA2* genes), Lynch syndrome (LS) (associated with *MLH1, MSH2, MSH6,* and *PMS2* genes), and FH (associated with *APOB* and *LDLR* genes)—are collectively identified as “Tier 1” conditions. For CDC Tier 1 conditions returned through MyCode, 87% (305/351) of patient-participants were unaware of their molecular diagnosis at the time of the genomic result (Buchanan et al., 2020). In another report, only 13% (7/55) of individuals with a *BRCA1/2* variant returned through MyCode had previously received clinical genetic testing that identified their molecular diagnosis (Buchanan et al., 2018). Among individuals with a *BRCA1/2* variant returned through MyCode, 51% (45/89 individuals) met National Comprehensive Cancer Network (NCCN) criteria for clinical testing, yet had no documentation of genetic testing or referral to genetic counseling (Manickam et al., 2018). For FH, none of the individuals meeting the clinical criteria for “definite” or “probable” FH diagnosis had previously undergone genetic testing (Buchanan et al., 2020). Importantly, not all these individuals with FH would have been identified using clinical screening criteria (Jones et al., 2021a).

#### Rate of Relevant Genetic Disease

In MyCode, 65% of individuals identified with a P/LP variant in one of the CDC Tier 1 conditions had a personal or family history relevant to the condition (Buchanan et al., 2020). Individuals identified with an FH variant had significantly increased odds of having general (odds ratio, 2.6) and premature coronary artery disease (odds ratio, 3.7) compared to individuals with high cholesterol but without a genomic variant (Abul-Husn et al., 2016). MyCode participants with a P/LP *BRCA1/2* variant were significantly more likely than participants without a *BRCA1/2* variant to have a history of breast cancer (odds ratio, 5.95) or ovarian cancer (odds ratio, 18.3) (Manickam et al., 2018). For ACM, although some of the 140 individuals with a P/LP variant were found to have a relevant clinical feature, the prevalence of EHR-recorded cardiac findings did not differ compared to matched controls without a P/LP variant (Carruth et al., 2019). Further phenotyping among 59 individuals with a P/LP ACM variant found that only 1 (2%) met a strict definition of a clinical diagnosis of ACM, though an additional 20 (34%) satisfied at least one ACM diagnostic criterion (Carruth et al., 2021).

#### Impact of Disclosure on Medical Management

Across CDC Tier 1 conditions, 70% of individuals eligible for condition-specific risk management engaged in at least one risk management procedure 1–3 years post-disclosure. However, uptake was highly variable between conditions and management procedures (Buchanan et al., 2020). For females without any prior cancer diagnosis who received a *BRCA1/2* result from MyCode, mammogram or breast MRI uptake was between 50%–92% and 11%–31% had a risk reducing salpingo-oophorectomy, depending on when the analysis was performed (Buchanan et al., 2018; Buchanan et al., 2020; Hao et al., 2020). Among individuals who received a P/LP result related to FH, nearly all had lipid testing post-disclosure, 51%–83% discussed their FH result with a clinician, and 38% had important changes to their treatment regimen (Jones LK et al., 2018). Specific to FH, we also reported an increase in adherence to important lipid lowering therapy from 64 to 77% post-disclosure and in another study reported on 3 individuals above the lipid control goal (LDL-C < 100 mg/dl) pre-disclosure who met goal after disclosure which prompted following appropriate risk management specific to FH (Jones LK et al., 2018). For clinicians, disclosure of an FH result through MyCode led to ordering of lipid testing and referral for evaluation in nearly all identified individuals (Jones LK et al., 2018).

FH is the only condition in which we have reported on multi-level barriers and facilitators to guideline-recommended care (Jones et al., 2020). Patients reported multiple barriers, including experiencing care gaps due to changing evidence, lack of insurance coverage for treatment, side effects related to treatments and other family or health demands that impeded them from managing their FH. They noted having an informed medical team facilitated their care (Jones et al., 2020). Medical management barriers reported by clinicians included lack of awareness of FH, busy clinics, and difficulty convincing patients to adhere to prescribed treatment plans. Having clear diagnostic criteria was identified as a facilitator of medical management for FH (Jones et al., 2020). These results have been used to guide implementation strategy development for programs to improve medical management and inform further research (NHLBI-funded grant R61HL161775) for FH in identified individuals.

#### New Clinical Diagnoses Post-Disclosure

Among 305 MyCode participants found to have a molecular diagnosis of a CDC Tier 1 condition, 41 (13%) were found to have a post-disclosure cancer diagnosis or diagnosis of FH-related features within 22 months from disclosure (Buchanan et al., 2020). Twenty-five (61%) of these diagnoses were determined to be attributed to the result being returned *via* MyCode (Buchanan et al., 2020). An early case series reported on three cases with *BRCA1/2* variants whose personal and family history did not meet genetic testing referral guidelines but were found to have early-stage *BRCA1/2*-related cancers after risk management prompted by disclosure of the genetic result (Buchanan et al., 2018). In studies of FH, none of the individuals with an FH variant detected through MyCode had a clinical diagnosis of FH recorded in the EHR prior to disclosure. After disclosure of a genetic risk result for FH, only 29% had the clinical diagnosis code for FH added to their problem list in their EHR (Jones LK et al., 2018). Of 59 individuals with follow-up for ACM, two individuals received new cardiomyopathy diagnoses and had implantable defibrillators for primary prevention placed (Carruth et al., 2021).

#### Cost and Cost-Effectiveness

In a study of cost-burden to the healthcare system, no statistically significant differences in healthcare utilization and average total costs of care between one-year pre- and post-disclosure of a *BRCA1/2* variant in MyCode patient-participants were found ($18,821 vs. $19,359, *p* = 0.76) (Hao et al., 2020). Modeling studies demonstrate that population-based DNA screening for HBOC in unselected women at age 30 is likely to be cost-effective (incremental cost-effectiveness ratio was $87,700/quality-adjusted life year) (Guzauskas et al., 2020), and cascade testing of first-degree relatives modestly improves clinical and economic value. In contrast, population-based DNA screening for LS may be cost-effective in younger patient populations, but the plausible range of cost-effectiveness was higher than that for HBOC, and depended to some degree on lower test and intervention costs (Guzauskas GF et al., 2022).

### Adoption

From July 2018 to June 2021, a total of 1,026 clinical DNA screening tests were ordered by 60 clinicians across the three pilot clinic sites in the clinical DNA screening pilot program. Of the 60 clinicians who ordered the DNA screening test at least once, 29 (48.3%) were attending physicians, 28 (46.7%) were medical fellows or residents, and 3 (5%) were advanced health practitioners (including certified nurse practitioners and physician assistants). Attending physicians generally ordered more tests than other types of clinicians (median [range]: 8 [1–532] tests ordered compared to 1 [1–21] ordered by medical residents or fellows, and 6 [1–17] ordered by advanced health practitioners).

### Implementation

Clinicians practicing at the pilot clinic sites were invited to participate in interviews about their early experience with the clinical DNA screening pilot program. Among the 14 interviewed clinicians, eight (57%) were male, and nine (64%) were attending physicians. Attending physicians who completed interviews had practiced medicine for an average of 17 years, with 11.8 of those years at Geisinger. Residents and fellows who completed interviews practiced medicine an average of 2.4 years, all of them at Geisinger. Interviewed clinicians had a range of experience with ordering the clinical DNA screening tests; seven (50%) had never ordered the test. These preliminary interviews provided insights into the ordering practices of the DNA screening test by clinicians at pilot clinic sites under the initial implementation conditions:

#### Motivation to Order Test

Clinicians who ordered the clinical DNA screening test communicated their motivation to empower and partner with patients and families to manage their health as “giving them that power to be able to make those decisions and walk them through that is very important” (ID14; 1 test ordered).

#### Test Utility

One clinician indicated not ordering the test for older patients due to perception of limited medical utility in that age demographic, stating “with my 90-year-olds … they’re really past the point where if they had the disease, you would know about it by now” (ID34; 18 tests ordered). Other low adopters expressed beliefs that DNA screening lacks evidence to support use compared to other routine screening tests.

“My impression, at this point, is it is [the yield of DNA screening] less than the screening test that we have for, you know, breast cancer and screening for colon cancer, things of that nature, but like I said, I’m not sure what the actual yield is, because I know a majority of my patients who were screened had no abnormalities” (ID53; 11 tests ordered).

Conversely, high-adopters compared the DNA screening test to other screening tests (e.g., mammograms and colonoscopy) saying, “I offer this test just the same way as I would a colonoscopy or emphasize the importance of any of the immunizations which may be age-appropriate for them. So, it is just part of the whole package that I talk about…” (ID23; >100 tests ordered).

#### Understanding Test Application

Some interviewed clinicians reported only ordering the DNA screening test when they suspected a genetic condition or if they desired a result for the patient more quickly than through research avenues, such as MyCode. This suggests some clinicians may have an unclear understanding of the purpose of using a screening test (the DNA screening test) in clinical practice and the purpose of diagnostic testing (the traditional indication-based testing process where patients could be referred to genetics clinic).

#### Implementation in Primary Care

All interviewed clinicians expressed favorable views about the process for ordering the clinical DNA screening test. They also endorsed the result disclosure model of having a genetic counselor disclose positive results using the MyCode GSC program processes and expressed the importance of providing patients with access to genetics professionals to explain result implications.

Some clinicians expressed questions related to who would cover costs for downstream testing or cascade testing of family members if a patient was found to have an actionable variant when discussing implementation in primary care. Logistics around time and clinic workflows in primary care were also noted stating “There’s alot of stuff that happens within a short 15–20 min visit, … a lot of physicians are already time crunched … this is just another one of those things that we need to do on top of that” (ID44; 2 tests ordered).

Finally, some interviewees recalled attending informational sessions for the pilot program while others reported learning about the test and how to order it only from other clinicians at their site. Therefore, future implementation strategies suggested by interviewees include standardized workflows for test ordering and results reporting, additional informational material for clinicians and patients, and recurring clinician training.

## Discussion

DNA-based population screening shows promise for improving population health but new methodologies, such as implementation science, are needed to understand its clinical utility from the rapidly growing evidence base and to facilitate the translation of effective DNA-based screening practices into clinical care (Murray et al., 2019; Williams, 2022). A key strength for this analysis is Geisinger’s commitment to innovation in exploring precision health approaches through the existence of multiple programs currently generating evidence (Carey et al., 2016; Schwartz et al., 2018; Schmidlen et al., 2019; Savatt et al., 2020). This study demonstrates a pragmatic analysis of outcomes derived from two DNA screening programs implemented at Geisinger for different purposes. We applied the RE-AIM implementation science framework to collectively analyze and report outcomes (Glasgow and Estabrooks, 2018). As more DNA-based population programs are being launched (Williams, 2022), this approach highlights the use of the RE-AIM framework to conduct a pragmatic program evaluation and demonstrates how the fields of genomics and precision health can utilize implementation science methods to capitalize on data generated from research and non-research programs implemented under real-world circumstances (Feero et al., 2018; Khoury et al., 2018; All of Us Research Program Investigators, 2019; Grzymski et al., 2020).

Results from the post-hoc Reach and Effectiveness evaluation of MyCode suggest most individuals at Geisinger approached are willing to participate in a research program that discloses health-related genomic information, and that DNA screening in this manner can positively impact the identification of genes and diseases tested when offered to unselected individuals. Results from the Adoption and Implementation evaluation of Geisinger’s pilot clinical DNA screening program suggest that clinicians will order the test for their patients and that broader implementation should include ongoing education opportunities and be aligned with current clinical workflows.

Evaluation of MyCode’s Reach as of August 2020 identified a reasonably high participation rate (68%), but also a need to better engage potential participants who reflect the full spectrum of diversity within the Geisinger population. While the population of central Pennsylvania is of primarily Northern European, non-Hispanic descent, MyCode participants have less diversity than the general Geisinger population. Potential opportunities for expanding the reach of MyCode include translation of consent into other relevant languages (English and Spanish currently available) and targeted engagement with underrepresented populations in our catchment area. Exploration of the barriers and needs of these populations is also an important next step to further ensure equitable access to precision care as these research programs are translated into the clinic. MyCode participants are also significantly more likely to have a Geisinger primary care provider and/or Geisinger health insurance coverage, suggesting that the Reach of a DNA-based population program could be greatest in a health system among those with an established patient-clinician relationship or where there are multiple opportunities to gain access to such screening throughout a system.

Evaluation of MyCode Effectiveness outcomes emphasized the potential for a research-based DNA screening program to improve health outcomes and highlighted Effectiveness gaps that remain to be studied. Further study of the clinical utility of screening for P/LP variants in the genes screened by MyCode other than those associated with HBOC, Lynch syndrome, FH, and ACM is indicated. Effectiveness studies from MyCode data are in process for several non-Tier 1 conditions, such as hereditary hemochromatosis, endocrine tumor syndromes, Long QT syndrome, and malignant hyperthermia, which should enrich our understanding of DNA screening in these conditions. Qualitative and quantitative evaluation of individual-level reactions to receiving genomic information in and across these conditions will further define the clinical and personal utility of DNA screening, as will studies addressing multi-level barriers and facilitators of post-disclosure medical management (clinician and system utilization of the result).

Additional cost-effectiveness analyses are underway for FH (Spencer et al., 2019) and continued modeling of integrated screening for all CDC Tier 1 conditions will inform decision making on reimbursement of DNA screening. We expect additional condition-specific gaps and cost analyses to be addressed as research capacity is increased to include individuals focused on other conditions and at different levels of the translational spectrum. To date, we have not reported on long-term health outcomes or improving adherence to recommended risk management at the individual-, clinician-, or system-level. Geisinger has only been disclosing results from MyCode since 2015 and the clinical DNA screening pilot program was formalized in 2018, therefore health outcomes and adherence data is currently limited and studies of interventions to impact adherence are just beginning. As MyCode continues to return results over the coming years, maintenance outcomes at the patient level related to DNA screening will be possible to analyze and report.

The clinical DNA screening program was used to assess early Adoption and Implementation outcomes, with more than 1,000 tests ordered as part of the clinical pilot. Qualitative interviews with clinicians who ordered and did not order the test identified a general acceptance of population DNA screening, with adopters finding the test ordering and result disclosure processes acceptable as currently implemented. Longitudinal data collection (both qualitative and quantitative) on adoption and implementation will be necessary to explore and demonstrate maintenance outcomes at the clinician and system level in the future.

Published literature demonstrates the importance of utilizing qualitative inquiry when reporting RE-AIM outcomes (Holtrop et al., 2018b). Our qualitative data on Implementation identified reasons clinicians interviewed did not order the test and several implementation strategies for iterative improvement in the clinical DNA screening pilot program. Ongoing education and other strategies to ensure clinician awareness and knowledge of processes could be instituted and evaluated to determine incremental improvement in test ordering and program implementation. Our early data from this pragmatic use of RE-AIM is providing guidance for implementation of a DNA screening program that fits the context of ambulatory care, thereby enabling sustainability, and is guiding the data collection approach and analyses that will inform precision health impact within the virtuous cycle of a learning healthcare system (Glasgow and Estabrooks, 2018; Glasgow et al., 2019).

The number of programs exploring the utility of DNA screening is growing rapidly (Williams, 2022), generating calls for harmonization of effectiveness data across programs and studies to improve the value of outcomes reported (Williams et al., 2018a). A few cross-program assessments of barriers and learnings have been reported from the funded IGNITE and eMERGE networks (Zebrowski et al., 2019; Wiesner et al., 2020; Sperber et al., 2021; Leppig et al., 2022). Similar cross-program evaluations could be conducted utilizing RE-AIM or other implementation science frameworks in combination to synthesize evidence (Reilly et al., 2020) from the myriad of other programs (Williams, 2022) being conducted in both research and non-research contexts but not connected to these large networks. Our work provides a blueprint for moving beyond the traditional reporting of intervention effectiveness alone by utilizing implementation science and the RE-AIM framework to report on additional framework outcomes of Reach, Adoption, Implementation, and Maintenance across multiple DNA screening programs designed for different purposes. This approach could accelerate learnings and reduce the research-to-practice gap in DNA-based population screening and have a broader public health impact. Furthermore, a harmonized approach will facilitate evaluation of key differences in programs, including funding sources, information returned, process of consent and return, and implementation processes and costs. This data will be critical if we are to rapidly learn from the growing number of research and clinical DNA screening programs and provide the evidence needed for broad implementation to ultimately realize the public health impact of DNA screening.

The harmonized assessment of RE-AIM domains can also help prevent DNA screening programs from creating unintended adverse consequences or exacerbating health disparities. Over 90% of the participants in MyCode were of self-reported non-Hispanic, European ancestry (Buchanan et al., 2020). Similarly, over 70% of the participants in the eMERGE III cohort, a large NIH-sponsored network researching genomic screening, were also self-reported (eMERGE Consortium, 2019). This lack of diversity in genomics research impedes our understanding of potential differences in outcomes across, and how to best tailor DNA screening for, diverse populations. Therefore, it is critical that the multiple precision health programs currently working to improve engagement with under-represented populations include assessment of implementation outcomes (Williams, 2022). To further address disparities and facilitate equity, recent recommendations include consideration of health equity through integration of other existing frameworks with RE-AIM evaluations to address this important contextual factor (Shelton et al., 2020). The Reach dimension includes assessment of representativeness, but more recent guidance specifically calls for assessment across subgroups involved, such as social determinants, rural or racial/ethnic populations, healthcare setting resources (high or low resourced), or literacy, to demonstrate who the program benefits and where inequities may continue to exist (Shelton et al., 2020).

### Limitations

While the existence of multiple precision health programs at Geisinger enabled these analyses, it is important to acknowledge the limitations inherent in collecting data within a single healthcare system. First, while not a limitation to our study, but one which could influence broader adoption, is Geisinger’s ability to implement a clinical DNA screening pilot program based on the pre-existing acceptance of the MyCode research initiative within the system. The broad recognition of the successes of this research program across our system may have facilitated clinician interviewees’ general acceptance of the program, regardless of whether they had ordered the test or not. Adoption and Implementation outcomes may be different in contexts where DNA screening is less salient to clinicians or in health systems naïve to genomics at the scale of MyCode. Secondly, our genomics programs were impacted by the COVID-19 pandemic. MyCode suspended all in-person consenting from March–December 2020, and while individuals could still consent to MyCode through electronic means, this mode currently does not yield a significant number of consents. Therefore, it is possible we would have demonstrated a higher participation rate if not for the pandemic. The COVID-19 pandemic also impacted additional implementation strategies for the clinical DNA screening pilot, in that all efforts to provide additional education and support for clinicians were suspended in pilot sites. Furthermore, while not specifically stated by the clinicians interviewed, the switch to virtual care in the ambulatory care setting under the stress of the pandemic may have limited the overall ordering of tests by clinicians. Therefore, results related to Adoption and Implementation must be interpreted within this specific context. Finally, this evaluation was based on the post-hoc, pragmatic use of RE-AIM, and as such, data collection/availability was limited to that which is accurate and practicable to extract from available program and clinical sources. The strength of this approach is that the results provide insight into outcomes under real-world conditions and identify areas where resources might be directed to either improve existing clinical data availability or to provide for resource-intensive data collection and analyses. As more programs for population-based genomic screening are piloted (Williams, 2022), studies could be prospectively designed and resourced to enable the evaluation of all RE-AIM dimensions from one or multiple genomic screening programs. Studies may also be designed to use RE-AIM in combination with other implementation science frameworks as appropriate (Reilly et al., 2020; Shelton et al., 2020).

## Conclusion

We applied the RE-AIM implementation science framework to conduct a pragmatic program evaluation to assess what two research and DNA screening pilot programs reveal that can inform future uptake of DNA-based population screening. We provide important evidence for such screening and through this approach of utilizing data from different programs relevant to each RE-AIM domain we identify remaining gaps necessary to address clinical utility, adoption, and implementation of programs in health care systems. This pragmatic approach of utilizing data from different programs most informative for each RE-AIM dimension will be important as more hospitals and health systems begin piloting their own DNA-based population screening programs.

## Data Availability

The original contributions presented in the study are included in the article/Supplementary Material, further inquiries can be directed to the corresponding author.
