# Supplementary material for: A RE-AIM Framework Analysis of DNA-Based Population Screening: Using Implementation Science to Translate Research Into Practice in a Healthcare System
Source: Front Genet. 2022 May 25;13:883073. doi: 10.3389/fgene.2022.883073 (PMC9174580; doi:10.3389/fgene.2022.883073)
Supplement: Supplementary file 1 [file Table1.docx]

Supplementary Material

# Supplementary Material 1. Interview Guide

**Population Health Interview Guide**

Thank you for agreeing to do this study with us. As a reminder, your participation in this study is voluntary. We expect this interview to last up to 30 minutes. Your responses will help us learn more about the DNA screening test at Geisinger. Please be honest with your answers. You can say whatever you want – nothing will hurt my feelings and nothing you say will have a negative effect on your employment status. Everything you tell me will be kept confidential. We will record this conversation, but the transcript from the conversation will not include any information that identifies you. Please remember: you can decline to answer any question and you may end the interview at any time.

Is it OK if I start recording now? (Utilize transcription service).

ALL

Ask all providers:

Utilizers

Ask only providers who **have** offered / ordered tests:

Ask only providers who have **not** offered / ordered tests:

Non-utilizers

**Interview Guide**

ALL

**Provider Demographics**

- Tell me about your role in Geisinger.
- How many years have you been practicing?
- How many years have you been at Geisinger?
- What is your experience with ordering genetic testing?
  - What type of genetic testing have you ordered?
- Now I’m just going to ask you a few questions about yourself. Are you comfortable sharing your age with me? If not, you can share whether you’re in your 30s, 40s, 50s, etc.
- Could you please tell me your gender and preferred gender pronouns?

ALL

**Impressions and Thoughts about Population Health DNA screening tests**

- Tell me about your experience with the DNA screening test that is orderable within Epic as the “Whole Exome Sequencing – Population Health Subset Test” (will be referred to as DNA screening test throughout).
  - Have you heard of the (Population Health) DNA screening tests?
    - IF NO, say the following to the clinician: The goal of this DNA screening test is to identify patients at increased risk for certain genetic conditions such as cancer and cardiac disease. This test is currently being piloted in select clinics to evaluate its utility in detecting disease earlier, allowing early intervention, better management, and improved outcomes.
  - How do you view this DNA screening test in relation to other screening tests you offer your patients?

**DNA Screening Test Information**

ALL

Overall impression of DNA screening test information sessions

- Do you recall attending any informational sessions for the DNA screening tests at your clinic?
  - - IF YES:
      - How was the [session that clinician attended] helpful (or, not helpful) for you to understand the test and when to consider offering it to your patients?
        - [if unsure/unclear] compared to other kinds of screening tests or genetic tests?
        - What went well with the information that you received?
        - What didn’t go well?
      - How well-equipped do you feel to offer the DNA screening test to your patients?
    - IF NO
      - How do you remember learning about ordering the DNA screening test?
      - Do you remember any informational materials related to the DNA screening test?
        - What can you tell me about them and how you used them?
        - What worked for you with these informational materials?
        - What didn’t work for you ?
      - How well-equipped do you feel to offer the DNA screening test to your patients?
      - What is your understanding of *when* to offer this test?
        - As compared to other kinds of genetic tests?

ALL

Provider input on future training

- What other resources for you or your patients would be helpful for you regarding the DNA screening test?
  - - IF clinician offers specific resources (e.g., flyers, goals), ask “Can you tell me how you see yourself using [resource].”
- How often, and in what format, do you think informational sessions on the test could be offered?

**DNA screening test utilization**

Categorizing providers as heavy vs. light utilizers of the DNA screening tests

Utilizers

- How often would you say you offer this test to your patients?
  - Approximately what percentage of patients?
  - How would you say that you use this test in your practice?
  - How do you value the test in helping you care for your patients?
  - Please tell me about the time it takes you to offer the test to your patients.
    - Ordering the test?
    - Discussing the results?
- How often would you say that patients accept/decline testing?
  - How do you typically discuss the test with your patients?
    - What do you say when offering?

Utilizers

Determining reasons for high vs. low utilization

- What factors would you say influence you when you think of offering the test to patients?
  - Is there anything about the patient that makes you think of ordering the test?

ALL

General barriers to ordering DNA screening tests

- What have you noticed keeps you from offering the test to your patients?
  - - Ordering the test?

Discussing the results?

- Tell me about any impact you have seen of the COVID-19 pandemic on your use of the DNA screening test?

Utilizers

**DNA screening test logistics**

General description of ordering process used for DNA screening tests

- Please describe your process when offering a test to one of your patients.
  - IF not already discussed: Tell me about what you say to patients when offering them the test.
- If you offer the test to a patient and they decline, do you document this in Epic, and if so, how is it documented?

Do you use standardized text?

- - Do patients provide a reason of declining, and is that documented?
  - Anecdotally, do you have a sense for why patients might decline?
  - If you have identified a type of patient who declines more frequently, how do you feel this impacts your willingness to offer the test?
    - Do you think some of your patient are declining because they have a result already, for example through MyCode or a clinical test?
- What do you think works for you when consenting / talking about the test with a patient?
  - What doesn’t work well when consenting / talking about a test?
- What do you think works for you when ordering a DNA screening test?
  - What doesn’t work for you when ordering a test?
- In the future, what are some ways that we can make this process better?

Utilizers

Characterization of DNA screening test SmartSet

- have you ever used the SmartSet created for the DNA screening test to put in the orders?
  - How often have you used the SmartSet?
  - What do you think of the SmartSet?
  - What works well for you when using the SmartSet?
  - What didn’t go well when using the SmartSet?
- If not, then how did you order the test?
- What influenced whether or not you used the SmartSet versus placing the order and documenting consent independently in the EHR?

Utilizers

**Follow-up with patients**

- How often do you discuss the test results with your patients?
- What influences whether you have such conversations with patients?
  - Does it matter if the results are positive or negative?
- What can you tell me about your conversations with patients after they have received the test results?
  - For those with a positive result, my understanding is that findings are first disclosed to the patient by a genetic counselor - do you feel that this model works effectively?
  - For those with negative results, my understanding is that results are disclosed to the patients by a letter – do you feel that this model works effectively?
  - What is your impression of how patient’s feel about this model?
  - What have your patients expressed to you about this model?
  - Has anyone had a good experience with this model?
    - A bad one?

Non-utilizers

**Understanding non-utilization of the DNA screening tests**

- Have your patients already received this test and/or a result of another genetic test?
- What are your thoughts about how much time it will take to implement the DNA screening test in your practice?
- What is your impression of the test ordering process?
  - In terms of how easy or difficult it may be?
  - How do you feel about the use of the SmartSet created for the DNA screening test?
  - In the future, what are some ways that we can make this process better?

ALL

**Other / concluding thoughts**

- Do you have any other thoughts about the DNA screening test that you want to share with me?
- Can you think of any other ways in which we can improve this test (e.g., the process of ordering it)?
- Thank you for this. Our ultimate goal is to offer this screening test to all patients – what are your thoughts about that?
  - What are your thoughts on how to make that happen?

*Note: if clinician requests information regarding the DNA screening tests, then make a note of their preferred contact information so that we can send them some materials on the PopHealth tests.

# Supplementary Material 2. Published Manuscripts Relevant to the Effectiveness of MyCode

| Article | Disease state | N | Study question | Screen positive detection rate of actionable genetic variants in unselected populations | Ascertainment of at-risk individuals via DNA screening compared to clinical ascertainment | Rate of relevant genetic disease | Impact of disclosure on medical management | New clinical diagnosis post-disclosure | Cost and cost effectiveness |
| --- | --- | --- | --- | --- | --- | --- | --- | --- | --- |
| Buchanan AH et al. *Genet Med*. 2021 (1) | CDC Tier 1 Conditions | 305 | To understand the impact of genomic screening on risk management and early detection in an unselected population |  | x | x | x | x |  |
| Kelly MA et al. *Am J Med Genet C Semin Med Genet.* 2021 (2) | ACMG Secondary Findings v2.0 List | 3,355 | Describe the MyCode variant screening pipeline, highlighting the benefits and unique challenges encountered when scaling and integrating this research-based process into a learning healthcare system | x | x |  |  |  |  |
| Jones LK et al. *Circ Genom Precis Med*. 2018 (3) | FH | 23 | To understand the impact on disease management and care patients seek after return of an FH genetic risk variant |  |  |  | x | x |  |
| Abul-Husn NS et al. *Science*. 2016 (4) | FH | 229 | To uncover and characterize novel variants, determine disease prevalence, and impact on clinical outcomes | x |  | x |  |  |  |
| Jones LK et al. *Coron Artery Dis*. 2021 (5) | FH | 117 | To determine statin benefits groups of individuals with pathogenic FH results |  | x |  |  |  |  |
| Jones LK, et al. *PLoS One*. 2020 (6) | FH | 50 | To evaluate patient and clinician barriers and facilitators to treat FH and explore solutions |  |  |  | x |  |  |
| Manickam K et al. *JAMA Netw Open*. 2018. (7) | HBOC | 267 | To identify and characterizes features of individuals with BRCA1/2variants in an unselected research cohort | x | x | x |  |  |  |
| Hao J et al. *J Pers Med*. 2020. (8) | HBOC | 59 | To determine whether there is a difference in health care utilization and costs following results disclosure, and estimate the uptake of risk management per National Comprehensive Cancer Network (guidelines within the first year of having a P/LP result returned |  |  |  | x |  | x |
| Buchanan AH et al. *Genet Med*. 2018. (9) | HBOC | 33 | To report the impact of a BRCA1/2screening program among individuals previously unaware that they were carrying a pathogenic variant–risk-management initiation and cancer diagnoses prompted by results disclosure |  | x |  | x | x |  |
| Carruth ED et al. *Circ Genom Precis Med.* 2019. (10) | ARVC | 140 | To characters phenotype associated with ARVC | x |  | x |  |  |  |
| Carruth ED et al. *Circ Genom Precis Med*. 2021. (11) | ACM | 92 | To report clinical findings from first 92 patients identified with P/LP desmosome variants | x |  | x |  | x |  |
| Guzauskas GF, et al. *JAMA Netw Open*. 2020. (12) | BRCA and Ovarian Cancer | N/A | To estimate the lifetime incremental incidence of HBOC and the quality-adjusted life years, costs, and cost-effectiveness of HBOC genomic screening in an unselected population  vs family history–based testing |  |  |  |  |  | x |
| Guzauskas GF, et al. *Genet Med*. *In press.* (13) | Lynch syndrome | N/A | To address these questions by estimating the cost-effectiveness of germline genetic screening among unaffected, unselected individuals in the U.S. population for LS heterozygote status followed by cascade genetic testing of first-degree family members |  |  |  |  |  | x |

Abbreviations: ACM, arrhythmogenic cardiomyopathy; ACMG, American College of Medical Genetics and Genomics; ARVC, arrhythmogenic right ventricular cardiomyopathy; CDC, Centers for Disease Control and Prevention; FH, familial hypercholesteremia; P/LP, pathogenic and likely pathogenic, HBOC, hereditary breast and ovarian cancer

# References

1. Buchanan AH, Lester Kirchner H, Schwartz MLB, Kelly MA, Schmidlen T, Jones LK, et al. Clinical outcomes of a genomic screening program for actionable genetic conditions. *Genet Med* (2020) 22(11):1874-82. Epub 2020/07/01. doi: 10.1038/s41436-020-0876-4. PubMed PMID: 32601386; PubMed Central PMCID: PMC7605431.

2. Kelly MA, Leader JB, Wain KE, Bodian D, Oetjens MT, Ledbetter DH, et al. Leveraging population-based exome screening to impact clinical care: The evolution of variant assessment in the Geisinger MyCode research project. *American journal of medical genetics Part C, Seminars in medical genetics* (2021) 187(1):83-94. Epub 2021/02/13. doi: 10.1002/ajmg.c.31887. PubMed PMID: 33576083.

3. Jones LK RA, Manickam K, Butry L, Lazzeri A, et al. Healthcare Utilization and Patients’ Perspectives After Receiving a Positive Genetic Test for Familial Hypercholesterolemia: A Pilot Study. *Circ Genom Precis Med* (2018) 11:e002146.

4. Abul-Husn N, Manickam K, Jones LK, Wright EA, Hartzel DN, Gonzaga-Jauregui C, et al. Genetic identification of familial hypercholesterolemia within a single U.S. health care system. *Science* (2016) 354(6319). doi: 10.1126/science.aaf7000.

5. Jones LK, Jefferson CR, Chen N, Murray MF. Genetic screening for familial hypercholesterolemia identifies patients not meeting cholesterol treatment guidelines. *Coron Artery Dis* (2021) 32(6):588-9. Epub 2021/01/05. doi: 10.1097/mca.0000000000000998. PubMed PMID: 33394692.

6. Jones LK, Sturm AC, Seaton TL, Gregor C, Gidding SS, Williams MS, et al. Barriers, facilitators, and solutions to familial hypercholesterolemia treatment. *PLoS One* (2020) 15(12):e0244193. Epub 2020/12/29. doi: 10.1371/journal.pone.0244193. PubMed PMID: 33362269; PubMed Central PMCID: PMCPMC7757879.

7. Manickam K, Buchanan AH, Schwartz MLB, Hallquist MLG, Williams JL, Rahm AK, et al. Exome Sequencing-Based Screening for BRCA1/2 Expected Pathogenic Variants Among Adult Biobank Participants. *JAMA Netw Open* (2018) 1(5):e182140. Epub 2019/01/16. doi: 10.1001/jamanetworkopen.2018.2140. PubMed PMID: 30646163.

8. Hao J, Hassen D, Manickam K, Murray MF, Hartzel DN, Hu Y, et al. Healthcare utilization and costs after receiving a positive BRCA1/2 result from a genomic screening program. *J Pers Med* (2020) 10(1):7.

9. Buchanan AH, Manickam K, Meyer MN, Wagner JK, Hallquist MLG, Williams JL, et al. Early cancer diagnoses through BRCA1/2 screening of unselected adult biobank participants. *Genet Med* (2018) 20(5):554-8. Epub 2017/12/21. doi: 10.1038/gim.2017.145. PubMed PMID: 29261187; PubMed Central PMCID: PMCPMC5930270 article.

10. Carruth ED, Young W, Beer D, James CA, Calkins H, Jing L, et al. Prevalence and Electronic Health Record-Based Phenotype of Loss-of-Function Genetic Variants in Arrhythmogenic Right Ventricular Cardiomyopathy-Associated Genes. *Circ Genom Precis Med* (2019) 12(11):e002579. Epub 2019/10/23. doi: 10.1161/circgen.119.002579. PubMed PMID: 31638835; PubMed Central PMCID: PMCPMC6876858.

11. Carruth ED, Beer D, Alsaid A, Schwartz MLB, McMinn M, Kelly MA, et al. Clinical Findings and Diagnostic Yield of Arrhythmogenic Cardiomyopathy Through Genomic Screening of Pathogenic or Likely Pathogenic Desmosome Gene Variants. *Circ Genom Precis Med* (2021) 14(2):e003302. Epub 2021/03/09. doi: 10.1161/circgen.120.003302. PubMed PMID: 33684294; PubMed Central PMCID: PMCPMC8284375.

12. Guzauskas GF, Garbett S, Zhou Z, Spencer SJ, Smith HS, Hao J, et al. Cost-effectiveness of Population-Wide Genomic Screening for Hereditary Breast and Ovarian Cancer in the United States. *JAMA Netw Open* (2020) 3(10):e2022874. Epub 2020/10/30. doi: 10.1001/jamanetworkopen.2020.22874. PubMed PMID: 33119106.

13. Guzauskas GF JS, Garbett S, Zhou Z, Spencer SJ, Snyder SR, Graves JA, Williams MS, Hao J, Peterson JF, Veenstra DL. Cost-effectiveness of Population-wide Genomic Screening for Lynch Syndrome in the United States. *Genet Med* (2022) In press.
